# *TERT* rs2736100 and *TERC* rs16847897 genotypes moderate the association between internalizing mental disorders and accelerated telomere length attrition among HIV+ children and adolescents in Uganda

**DOI:** 10.1186/s12920-020-00857-z

**Published:** 2021-01-06

**Authors:** Allan Kalungi, Eugene Kinyanda, Jacqueline S. Womersley, Moses L. Joloba, Wilber Ssembajjwe, Rebecca N. Nsubuga, Pontiano Kaleebu, Jonathan Levin, Martin Kidd, Soraya Seedat, Sian M. J. Hemmings

**Affiliations:** 1 Department of Psychiatry, Stellenbosch University, Cape Town, South Africa; 2 Mental Health Section, MRC/UVRI and LSHTM Uganda Research Unit, Entebbe, Uganda; 3 Department of Medical Microbiology, Makerere University, Kampala, Uganda; 4 Department of Psychiatry, College of Health Sciences, Makerere University, Kampala, Uganda; 5 South African Medical Research Council/Stellenbosch University Genomics of Brain Disorders Research Unit, Faculty of Medicine and Health Sciences, Stellenbosch University, Cape Town, South Africa; 6 School of Biomedical Sciences, College of Health Sciences, Makerere University, Kampala, Uganda; 7 Statistics and Data Science Section, MRC/UVRI and LSHTM Uganda Research Unit, Entebbe, Uganda; 8 MRC/UVRI and LSHTM Uganda Research Unit, Entebbe, Uganda; 9 School of Public Health, University of Witwatersrand, Johannesburg, South Africa; 10 Centre for Statistical Consultation, Department of Statistics and Actuarial Sciences, University of Stellenbosch, Cape Town, South Africa

**Keywords:** Internalizing mental disorders, Telomere length attrition, *TERT* rs2736100, *TERC* rs16847897, HIV+ children and adolescents, Uganda

## Abstract

**Background:**

Internalizing mental disorders (IMDs) (depression, anxiety and post-traumatic stress disorder) have been associated with accelerated telomere length (TL) attrition; however, this association has not been investigated in the context of genetic variation that has been found to influence TL. We have previously reported an association between IMDs and accelerated TL attrition among Ugandan HIV+ children and adolescents. This study investigated the moderating effects of selected single nucleotide polymorphisms in the telomerase reverse transcriptase gene (*TERT*) (rs2736100, rs7726159, rs10069690 and rs2853669) and the telomerase RNA component gene (*TERC*) (rs12696304, rs16847897 and rs10936599) on the association between IMDs and TL, among Ugandan HIV+ children (aged 5–11 years) and adolescents (aged 12–17 years).

**Results:**

We found no significant interaction between IMDs as a group and any of the selected SNPs on TL at baseline. We observed significant interactions of IMDs with *TERT* rs2736100 (*p* = 0.007) and *TERC* rs16847897 (*p* = 0.012), respectively, on TL at 12 months.

**Conclusions:**

*TERT* rs2736100 and *TERC* rs16847897 moderate the association between IMDs and TL among Ugandan HIV+ children and adolescents at 12 months. Understanding the nature of this association may shed light on the pathophysiological mechanisms underlying advanced cellular aging in IMDs.

## Background

Human immunodeficiency virus-positive (HIV+) children and adolescents suffer a considerable burden of internalizing mental disorders (IMDs) (namely, depression, anxiety and post-traumatic stress disorder) [1–3]. Studies undertaken both in the developed (Europe and the United States) and developing world (sub-Saharan Africa) among HIV+ children and adolescents have documented rates of major depressive disorder of between 12.7 and 40% [2–8], and rates of anxiety disorders of between 9 and 32.2% [1–3, 6]. For IMDs combined, rates of between 12 and 27% have been documented in Uganda and South Africa, respectively [1, 9]. Among people living with HIV/AIDS, IMDs have been associated with a number of other negative outcomes, including accelerated cellular aging [10], faster HIV disease progression [11, 12], poor adherence to medication [12, 13], risky sexual behavior [13, 14], poor linkage to care for newly diagnosed HIV+ persons [15], increased HIV transmission (through the promotion of HIV drug resistance) [16] and impaired academic and social functioning [13].

While considerable research has investigated the psychosocial risk factors for IMDs among HIV+ children and adolescents, there is a paucity of research on biological risk factors, including genetic factors. Recent genome-wide association (GWAS) studies have identified loci for depression [17], anxiety disorders [18] and post-traumatic stress disorder (PTSD) [19, 20], providing evidence for the role of genetic variation in the etiology of IMDs. Nevertheless, the etiology of IMDs, and the biochemical and molecular contributions in particular, are largely unknown.

IMDs are associated with increased mortality and age-related diseases, such as cancer, heart and cardiovascular disease [21–24]. IMDs are also highly comorbid with both psychiatric and somatic disorders, including those associated with advanced aging [25]. Depression has, for example, been reported to be associated with chronic diseases (e.g., type 2 diabetes mellitus and cardiovascular disease) [25, 26], as well as chronic inflammation [27]. In addition, higher mortality rates have been reported among patients with mental disorders (e.g., depression and other affective disorders) compared to the general population, with this mortality mainly due to the same age-related diseases, such as cancer, cardiac and cerebrovascular disease [22–24].

Several studies have investigated the association between telomeres, the protein-bound deoxyribonucleic acid (DNA) repeat structures at the ends of chromosomes, and IMDs [28]. Telomeres are important in protecting chromosomes from fusing together during mitosis, thus preventing loss of genetic data [29, 30]. They shorten progressively with each cell division, eventually leading to DNA damage responses, replicative senescence, or programmed cell death [31]. Since telomeres shorten with each cycle of cell division, telomere length (TL) provides a marker of biological aging [32]. Evidence for the role of TL among patients with IMDs and comorbid age-related diseases may offer insights into a novel potential mechanism for the excess morbidity and mortality associated with IMDs [33].

Shorter TL has been reported among patients with depression [34–41], anxiety disorders [34, 42, 43] and PTSD [44–47]. However, there has been comparatively little research into the relationship between TL and IMDs in the context of HIV infection, which has itself been associated with TL attrition [48, 49]. Shortening of telomeres in seropositive individuals may be due to HIV infection-associated increases in inflammatory and oxidative processes, both of which drive TL attrition [50, 51] or due to the effect of some nucleoside analogue reverse transcriptase inhibitors [50]. Furthermore, HIV-associated illness and stigma can act as a chronic psychological stressor to further drive TL attrition [52, 53].

We previously found evidence to suggest accelerated TL attrition that was driven by IMDs among Ugandan HIV+ children and adolescents [10]. There is, however, a dearth of data on the mechanisms by which IMDs lead to TL attrition. IMDs act as chronic stressors [54, 55], producing long-lasting biological adaptations that could potentially explain TL attrition due to IMDs [56]. Specifically, chronic stress can exert long-lasting effects on the hypothalamic–pituitary–adrenal (HPA) axis, such that previous experience of stress may prime a heightened response on subsequent stressor exposure [57]. Chronic stress also increases inflammatory signaling, which may in turn produce a pro-oxidative environment. Both inflammation and oxidative stress have been negatively associated with TL [51].

TL is partially heritable, with heritability estimates ranging between 44 and 80% [58, 59]. Therefore, genetic variation may contribute to telomere maintenance [60] and this genetic variation may confer risk for accelerated TL attrition. TL is maintained by telomerase, a catalytic enzyme with a protein component encoded by telomerase reverse transcriptase gene (*TERT*) and an RNA template component encoded by telomerase RNA component gene (*TERC*). Together, these act to add small DNA repeat segments to the end of chromosomes, thus counteracting TL attrition [30, 61]. A large genome-wide meta-analysis of 37,684 individuals found that *TERT* and *TERC* were amongst several loci found to influence mean TL [60], suggesting that genetic variation in these telomerase components has functional implications on TL.

The *TERC* rs12696304 *G*-allele and rs16847897 *C*-allele have been found to be associated with shorter TL by a candidate gene study on Swedish samples and a GWAS study on British samples [62, 63]. In addition, the *TERT* rs2736100 *C*-allele has been associated with shorter TL [60], while *TERT* rs7726159 *AA* genotype was associated with longer TL by a candidate gene study using White European samples [64].

We have previously determined an association between IMDs and TL in a sample of Ugandan HIV+ children and adolescents [10]. Specifically, TL was significantly longer in IMDs cases at baseline but did not differ from control TL at 12-month follow-up, suggesting that TL shortening over the one-year period was greater in participants with IMDs. Given that *TERT* and *TERC* are involved in TL maintenance [30], the present study investigated whether genetic variation in *TERT* (rs2736100, rs7726159, rs10069690 and rs2853669) and *TERC* (rs12696304, rs16847897 and rs10936599) moderated the association between IMDs and baseline and 12-month TL. Having observed that IMDs were driving accelerated TL attrition in the previous study [10], we modelled IMDs as the independent variable and TL as the dependent variable. We thus assessed for the interaction between IMDs and *TERT* and *TERC* on TL.

## Methods

### Study design

This case–control study was carried out in children (aged 5–11 years) and adolescents (12–17 years). A total of 368 cases with any internalizing mental disorder (IMD) and 368 age- and sex-matched controls were included. Both cases and controls were Ugandans. This study was nested within the previously described CHAKA study [1, 65], which enrolled 1339 HIV+ children and adolescents (855 children and 484 adolescents) in Uganda. This study complied with the Code of Ethics of the World Medical Association (Declaration of Helsinki). The CHAKA study obtained ethical and scientific clearance from the Uganda Virus Research Institute (UVRI) Science and Ethical Committee (#GC/127/15/06/459) and the Uganda National Council of Science and Technology (# HS 1601). The present study obtained approval from the Higher Degrees Research & Ethics Committee, School of Biomedical Sciences, College of Health Sciences, Makerere University (# SBS 421) and the Health Research Ethics Committee of Stellenbosch University (#S17/09/179). Study subjects who were diagnosed with significant psychiatric problems were referred to mental health units at Entebbe and Masaka government hospitals.

### Study population

Study subjects were recruited from two HIV clinics in urban Kampala (Joint Clinic Research Centre (JCRC) and Nsambya Home Care) and three HIV clinics in rural Masaka (The AIDS Support Organization (TASO), Kitovu Mobile Clinic and Uganda Cares). All study subjects were on anti-retroviral therapy (ART).

### Selection of cases and controls

Cases were all participants in CHAKA who met criteria for diagnosis of any IMD at baseline as per the diagnostic and statistical manual of mental disorders-edition 5 (DSM-5) referenced Child and Adolescent Symptom Inventory-5 (CASI-5) [66]. All cases at baseline were ascertained, and the cases were then stratified by site (one of two sites), sex, age category (one of three categories) and socio-economic status (SES) (one of three SES categories). This resulted in a total of 36 strata (2 × 2 × 3 × 3). In each stratum the number of cases was ascertained (e.g. for males in site 1 in the youngest age category and the lowest SES group there were 9 cases). An equal number of controls (HIV+ children and adolescents without any psychiatric disorder) were then randomly sampled from the stratum concerned (so for males in site 1 in the youngest age category and the lowest SES group we sampled 9 controls), thus the controls were frequency matched to the cases on site, sex, age and SES.

### Inclusion and exclusion criteria

*Inclusion criteria*: (1) HIV-infected outpatients, registered with the HIV Clinics at JCRC or Nsambya Home Care at the Kampala study site and TASO, Kitovu mobile or Uganda Cares clinic at the Masaka site; (2) aged between 5 and 17 years at the time of enrolment; (3) conversant in English or Luganda, the language into which the assessment tools were translated; and (4) able to provide written informed consent/assent. Cases were HIV+ children and adolescents who had any depressive disorder (depression or dysthymia [persistent depressive disorder]), anxiety disorder or PTSD. Controls were age- and sex- matched HIV+ children and adolescents without any psychiatric disorder. Persistent IMDs were baseline cases that remained cases at 12 months while remitted ones were baseline cases that no longer qualified for a diagnosis at 12 months. *Exclusion criteria*: (1) seriously ill and unable to understand study procedures; and (2) any other psychiatric disorder other than the IMDs listed above.

### Procedures

As part of the CHAKA study, children and assenting adolescents, as well as their parents/caregivers, were interviewed using a structured questionnaire. The questionnaire included, amongst others, socio-demographic characteristics and depression, PTSD and anxiety modules of the DSM-5 [66]. The CASI-5 was administered by trained psychiatric nurses or psychiatric clinical officers at two time points (baseline and 12 months). The CASI-5 lists the symptoms of a wide range of psychiatric disorders including MDD, generalized anxiety disorder, PTSD and attention-deficit/hyperactivity disorder among others. Individual CASI-5 items are rated on a four-point frequency of occurrence scale ranging from never (0) to very often (3). Though there are several CASI-5 scoring algorithms, in the present study we used symptom count cut-off scores, which reflect the prerequisite number of symptoms for a clinical diagnosis. At each study visit, 4 ml of blood from each study participant was collected via venipuncture into an EDTA vacutainer and subsequently stored at − 80 °C pending DNA extraction for genetics analyses.

#### Determination of genotypes for selected polymorphisms in telomerase reverse transcriptase gene and telomerase RNA component gene

DNA samples were genotyped for each selected SNP in each of *TERT* (rs2736100, rs7726159, rs10069690 and rs2853669) and *TERC* (rs12696304, rs16847897 and rs10936599), using a kompetitive allele-specific PCR (KASP) assay (LGC, Middelsex, United Kingdom). This genotyping chemistry allowed for bi-allelic discrimination of SNPs. The genotyped SNPs in each of *TERT* and *TERC* were analyzed for linkage disequillibrium (LD) by creating an LD map using the default Gabriel LD [67], implemented in Haploview software, version 4.2 [68]. For SNPs that were in LD, haplotypes were generated using the Haploview software.q

### Power of the study

Using results from a study by Epel et al*.* [69], post hoc power calculations (described in [10]) indicated that our study was well-powered with 83.8% power to detect at least 5% reduction in mean TL between cases and controls. For the interaction analysis, assuming a type I error rate of 0.05, zero correlation of the outcome between cases and controls, 1.6 odds ratio of exposure in cases relative to controls, a 0.055 probability of exposure among controls and a 1:1 case: control ratio for 368 cases, our interaction analyses achieved a power of 87.5%.

### Statistical methods

Statistical analyses were conducted using Stata 15 (StataCorp, TX, USA). Socio-demographic characteristics (including socio-economic status) were compared between cases and controls. Socio-economic status (SES) was generated from a scale of 9 household items owned (car, motorcycle, refrigerator, electricity, bicycle, radio, telephone, cupboard and flask). Each item was weighted in the respective order, a car carrying a maximum weight of 9 and a flask a minimum weight of 1. A total score of items was generated, whose median cut-off of 13 was used to classify low and high SES. A score less than 13 was classified as low SES, while that greater than 13 was classified as high SES. Our study group [70] has previously used household items as a measure of SES in rural settings of Uganda. A *t*-test was used to compare CD4 counts between cases and controls to account for any disparity in HIV disease progression.

Associations between the different socio-demographic factors and TL were tested using one-way analysis of variance (ANOVA) to determine potential confounders. Independent sample *t*-tests were used to assess the association between IMDs and TL both at baseline and 12 months. *TERT* and *TERC* genotypes were assessed for HWE using a likelihood ratio test. The genotypes were not validated as genotyping was done by a service provider using an automated SNP genotyping array. One-way ANOVA was used to assess for the association between each of the investigated SNPs or haplotype with baseline and 12 months TL, as well as the TL change. A likelihood ratio test was used to assess for interaction between each of the polymorphisms and IMDs on TL both at baseline and 12 months controlling for age and sex, with Bonferroni corrections for multiple testing. The likelihood ratio test was also used to assess for interaction between the generated *TERT* rs2736100-rs7726159 haplotypes and IMDs on TL both at baseline and 12 months controlling for age and sex as well. To perform a Bonferroni correction, the *p* value threshold of 0.05 was divided by 2 (number of separate tests) to yield a corrected threshold *p* value of 0.025. These interactions were performed on all the explanatory variables even without observing significant main effects in order to rule out the possibility of cross over interaction where significant interactions may be observed for non-significant main effects. For significant interactions, mean TL at 12 months was plotted against genotypes to elucidate the nature of the interaction terms. Where required, 95% confidence intervals were calculated.

## Results

No significant differences were observed when socio-demographic variables were compared between case and control participants (Table 1).

**Table 1:** Distribution of socio-demographic factors in cases and controls

Variable (n)	Case n (%)	Control n (%)	*p* value
Sex			*p* = 0.111
Male (342)	160 (43.6)	182 (49.5)	
Female (393)	207 (56.4)	186 (50.5)	
Site			*p* = 0.941
Urban (415)	208 (56.5)	207 (56.3)	
Rural (321)	160 (43.5)	161 (43.7)	
Age			*p* = 0.374
5–11 years (389)	202 (57.6)	187 (54.2)	
12– 17 years (307)	149 (42.4)	158 (45.8)	
Education level			*p* = 0.371
No formal education (13)	9 (2.5)	4 (1.1)	
Primary (648)	323 (88.0)	325 (88.8)	
Secondary (72)	35 (9.5)	37 (10.1)	
Socio-economic status			*p* = 0.459
Low (332)	171 (46.5)	161 (43.8)	
High (404)	197 (53.5)	207 (56.2)	
Mean CD4 count at baseline	947.04	944.02	*p* = 0.939

CD4: cluster of differentiation 4; primary = 0–7 years of formal education; secondary = 8–14 years of formal education; low socioeconomic status = 0–13; high socio-economic status =  > 13 (see statistical methods section). All numbers that do not add up were due to missing data

The genotypes for all the selected SNPs were in Hardy–Weinberg equilibrium (HWE) (Table 2). None of *TERC* SNPs were in linkage disequilibrium (LD). However, *TERT* rs7726159 and rs2736100 were in LD, resulting in *CT*, *CG* and *AG* haplotypes (see Additional file 1: S1 and S2). The genotype frequencies for all the selected SNPs and the *TERT* rs2736100-rs7726159 haplotype freqencies are shown in Additional file 1: Table S1. The association between each investigated SNP and TL are shown in Table 2 below. None of the selected SNPs or the *TERT* rs2736100-rs7726159 haplotypes associated with baseline or 12 months TL or TL change; however, *TERC* 16847897 significantly associated with 12-month TL (Table 2).

**Table 2:** *p *values for Hardy–Weinberg equilibrium and association of each selected single nucleotide polymorphism and the generated haplotypes with telomere length

Single nucleotide polymorphism	*p* value of association with			HWE *p* value
	Baseline TL	12 months TL	TL change	
*TERC* rs16847897	0.650	0.014	0.393	0.461
*TERC* rs12696304	0.861	0.991	0.981	0.443
*TERC* rs10936599	0.406	0.142	0.510	1
*TERT* rs2736100	0.300	0.366	0.581	0.091
*TERT* rs2853669	0.438	0.507	0.591	0.798
*TERT* rs7726159	0.590	0.855	0.802	0.882
*TERT* rs10069690	0.567	0.831	0.802	0.274
*TERT rs2736100-rs7726159*	0.331	0.724	0.725	N/A

HWE: Hardy–Weinberg equilibrium; *TERC*: telomerase RNA component gene; *TERT*: telomerase reverse transcriptase gene; *TERT* rs2736100-rs7726159: *TERT* rs2736100 and rs7726159 haplotype; N/A: not applicable

### Moderating effects of single nucleotide polymorphisms in telomerase reverse transcriptase gene and telomerase RNA component gene on the association between internalizing mental disorders and telomere length

**Fig. 1:**
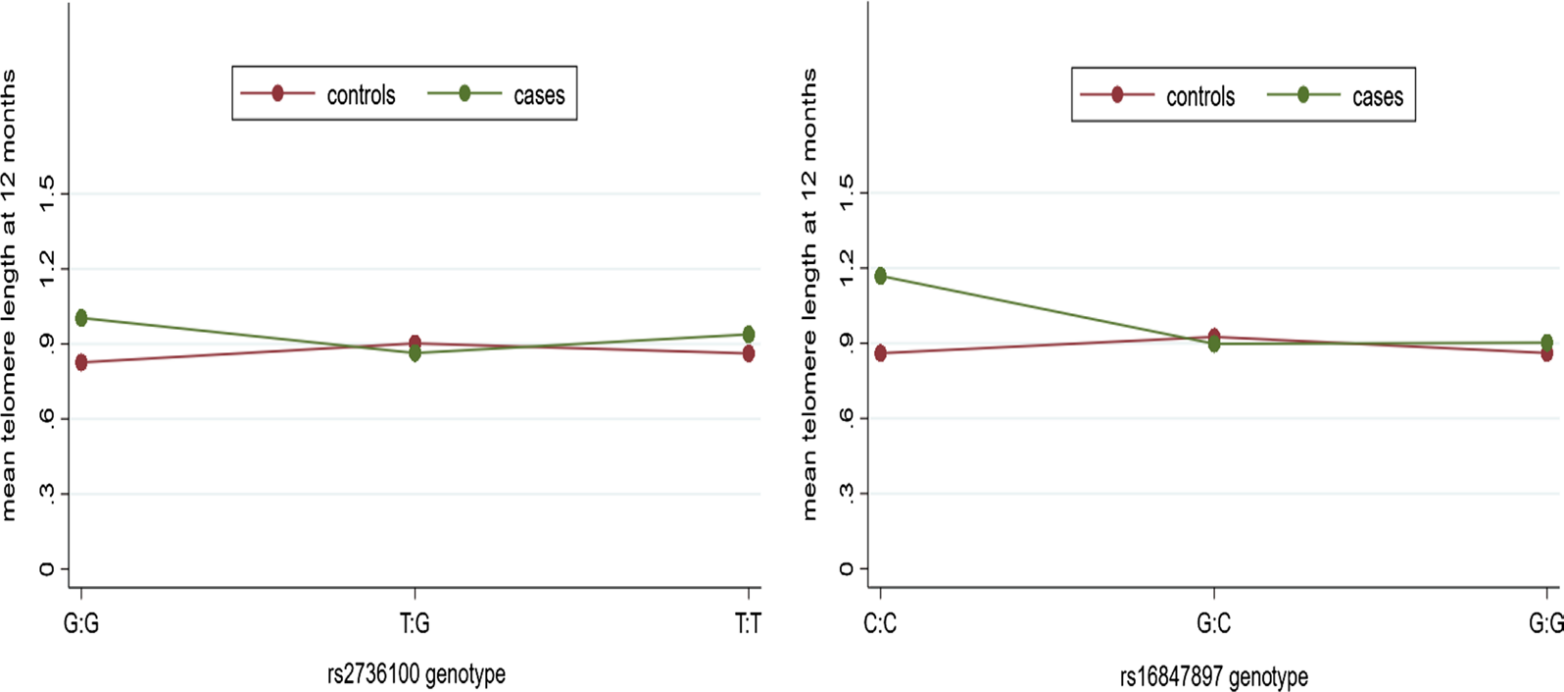
Mean TL between cases and controls by genotype for *TERT* rs2736100 and *TERC* rs16847897 at 12 months. For *TERT* rs2736100 mean TL was significantly different between cases and controls, but only in individuals with the *GG* genotype while for *TERC* rs16847897, mean TL was significantly different between cases and controls but only in individuals with *CC* genotype

We found no moderating role for any of the selected SNPs and on the association between IMDs and TL at baseline (Table 3). We did find that *TERT* rs2736100 and *TERC* rs16847897 significantly moderated the association between IMDs and TL at 12 months (*p* = 0.007 and *p* = 0.012 respectively) (Table 3). For *TERT* rs2736100, mean TL was longer among cases as compared to controls, but only in those individuals with the *GG* genotype (n = 139; 82 cases, 57 controls) (Fig. 1). No significant difference in TL was observed between cases and controls who possessed either the *TG* (n = 330) or *TT* (n = 201) genotypes (Fig. 1). For *TERC* rs16847897, mean TL was longer in cases compared to controls, but only in individuals with the *CC* genotype (n = 44; 27 cases, 17 controls) (Fig. 1). No significant difference in TL was observed between cases and controls who possessed either the *GC* (n = 265) or *GG* (n = 373) genotypes (Fig. 1). None of the *TERT* rs2736100-rs7726159 haplotypes significantly moderated the association between IMDs and TL at either baseline (*p* = 0.379) or 12 months (*p* = 0.418).

**Table 3:** Two-way analysis of variance for the interaction of internalizing mental disorders with rs2736100 and with s16847897 on telomere length at 12 months

SNP	Obs	Variable	F	Bonferroni corrected *p* value (α = 0.025)
Baseline				
rs2736100	596	IMDs	15.52	< 0.001
		rs2736100	0.98	0.375
		IMDs * rs2736100	1.15	0.318
rs16847897	597	IMDs	2.53	0.112
		rs16847897	0.37	0.692
		IMDs * rs16847897	1.79	0.168
12 months				
rs2736100	511	IMDs	6.03	0.014
		rs2736100	0.44	0.645
		IMDs * rs2736100	4.95	0.007*
rs16847897	515	IMDs	7.25	0.007
		rs16847897	3.00	0.050
		IMDs * rs16847897	4.44	0.012*

SNP: single nucleotide polymorphism; Obs: number of observations; IMDs * rs2736100: interaction of internalizing mental disorders with *TERT* rs2736100 on relative telomere length; IMDs * *TERC* rs16847897, interaction of internalizing mental disorders with rs16847897 on relative telomere length; *significant *p* value for the interaction term

## Discussion

Using the same samples of Ugandan HIV+ children and adolescents, we previously found that TL was longer at baseline in cases with IMDs, but that this difference did not remain 12 months later [10]. The present study built on those results and investigated whether selected polymorphisms within *TERT* and *TERC* interacted with IMDs to influence baseline and 12 months TL among the study participants. We found that *TERT* rs2736100 and *TERC* rs16847897 significantly moderated the association between IMDs and TL at 12 months. To our knowledge, this is the first sub-Saharan African study to investigate these interactions among HIV+ children and adolescents.

Telomeres are maintained by the telomerase enzyme, an enzyme whose catalytic protein component and RNA template is encoded by *TERT* and *TERC*, respectively [30, 61]. Given the role that TERT and TERC play in the structure of telomerase [71, 72], variations in genes encoding these components could influence TL. However, none of the investigated polymorphisms significantly influenced the association between IMDs and TL at baseline. We found a significant interaction between IMDs and *TERT* rs2736100 and *TERC* rs16847897 on TL at 12 months. This interaction points to a possible direct influence of IMDs on TL shortening. It is possible that chronic pathophysiological processes characteristic of IMDs, including inflammation, oxidative stress and HPA axis dysregulation [73], produced a cumulative burden over the one-year study period, with the resultant increase in allostatic load driving TL attrition. Plots of the interaction between IMDs and TL reveal that reductions in 12-month TL in cases are limited to participants carrying the *TERT* rs2736100 *GT or TT* genotype. Similarly, for *TERC* rs16847897, the *GC* or **GG** genotypes are associated with reduced TL at 12 months. These genotypes appear to accelerate TL attrition. However, it is hard to tease out the true effects of the genotypes on TL attrition in the present study, since the reduction in TL after 12 months was strongly significant in both cases and controls (*p* < 0.001 respectively), possibly due to the short 12 month period. Future studies with a longer study period are required to confirm our findings.

Although the functionality of these two SNPs is not well known, data from previous studies strongly suggest their involvement in disease-associated TL attrition (sporadic idiopathic pulmonary fibrosis), as well as pathophysiological mechanisms, such as inflammation, that are relevant to IMDs [74–76]. *TERT* rs2736100 is located within intron 2 of *TERT*, a position that has been described to be a putative regulatory region [77]. *TERT* rs2736100 has also been reported as a critical factor in TERT synthesis and activation [63]. *TERT* rs2736100 has previously been associated with diseases characterized by TL attrition, including lung cancer [78] and sporadic idiopathic pulmonary fibrosis [75, 76, 79]. TERT has also been reported to interact with natural factor kappa B (NF-κb) p65, where it activates NF-κb and increase metalloproteinases in cancer cells [74]. On the other hand, *TERC* 16847897 has also been reported as locus that could probably regulate TL. In line with findings of the present study, each copy of the *TERC* rs16847897 major *G*-allele (*CG* or *GG*) was associated with shorter mean TL in a Han Chinese population [80]. However, *TERC* rs16847897 *CC* genotype has also been associated with both shorter TL and lower TERT levels [81]. We have no direct explanation for this discrepancy. However, it is important to note that our findings result from a longitudinal interaction of the SNP with IMDs. The nature of this interaction needs to be elucidated in order to draw conclusions.

*TERT* rs2736100 and *TERC* rs18647897 loci appear to have regulatory functions and require further study. Although the nature of the interaction between IMDs and *TERT* and *TERC* on TL is not known, IMDs have been associated with increased oxidative stress [82, 83] and inflammatory markers, such as C-reactive protein and the pro-inflammatory cytokines interleukin-6 and tumor necrosis factor alpha [84, 85]. Oxidative stress leads to TL shortening through the inhibition of telomerase activity [73, 86, 87]. We hypothesize that IMD-related increases in oxidative stress and inflammatory responses produce a cumulative pathophysiological burden. The effect of this on TL may potentially be influenced by genetic variation in *TERT* and *TERC*.

Our study presents with limitations which deserve mention. First, the relationships between TL, IMDs and HIV are complex and, to a degree, reciprocal. HIV/AIDS may shorten TL via both infection-related pathophysiological processes (inflammation and oxidative stress), and its capacity to act as a chronic psychological stressor [52, 53]. Our study participants were also on ART, with some regimens, such as Effavirenz, reported to have psychiatric side effects [88]. Second, as we did not include an HIV- control group in our study, we can only speculate on the external validity of our findings. Nevertheless, since our results indicate that IMDs drive accelerated TL attrition [10], the SNP x IMD interactions observed in this study are potentially relevant to other populations. Further research would be required to investigate whether these findings are generalizable to Ugandan youths in the general population as well as other youth populations.. Third, we defined our cases as those individuals diagnosed with depressive disorder, any anxiety disorder or PTSD. The inclusion of PTSD (n = 60) in this sample is contentious, as the disorder has recently been excluded from the categorization of anxiety disorders in the DSM-5 [89]. This may have affected our findings. In order to elucidate the independent contribution of each particular disorder, future studies should analyze each IMD separately. Fourth, we did not control for population stratification at analysis since the study participants belonged to the Bagandan population group for which principal components analysis on GWAS data of a sample of over 4,000 individuals has shown that they are genetically similar, as principal components 1 and 2 have been reported to have explained only 0.3% and 0.1% of the genetic variation in a Bagandan general population cohort [90]. However, there is a possibility that some participants may not have been Baganda although they identified as Baganda and their inclusion could have caused population admixture that could have led to spurious results. Future studies should endeavor to control for population stratification.

Despite the limitations, our study has strengths that are worth mentioning. First, the study sample size was large enough (368 cases and 368 controls) to allow a sufficient power of greater than 80% for the association between IMDs and TL. Second, we measured TL at baseline and 12 months, a longitudinal aspect that allowed us to assess for causation. Third, TL undergoes a period of rapid attrition in the first five years of life, which is followed by relative stability until young adulthood [91, 92]. As our study participants ranged in age from 5 to 17 years and thus fell into this period of expected TL stability, the differences we identified are more likely to reflect valid influences of SNPs and IMDs, as opposed to chronological age effects.

## Conclusions

We observed that *TERT* rs2736100 and *TERC* rs16847897 produce effects on 12-month TL in Ugandan HIV+ children and adolescents diagnosed with IMDs. The mechanisms through which *TERT* and *TERC* SNPs interact with IMDs to influence TL are not known. Understanding these mechanisms may help to unravel the biochemical processes that take place following onset of IMDs, which may aid the discovery of new drugs or drug targets for better management of these disorders. Furthermore, these mechanisms may also provide insight into the biochemical processes that underlie the comorbidity between IMDs, age-related conditions and metabolic diseases. Future functional studies are required to understand how *TERT* and *TERC* SNPs moderate the association between IMDs and accelerated TL attrition.

## Supplementary Information

**Additional file 1**. **Figures S1** and **S2** show the linkage disequilibrium map of* TERT* and* TERC* single nucleotide polymorphisms respectively. **Table S1** shows the genotype and aplotype frequencies for* TERT* and* TERC* single nucleotide polymorphisms.

## Data Availability

All information gathered about study subjects and their samples is confidential, with access limited to the research team. However, upon request, data from the MRC/UVRI and LSHTM Uganda Research Unit is currently accessed under a data sharing policy via: http://www.mrcuganda.org/sites/default/files/publications/MRC_UVRI_Data_sharing_policyDecember2015.pdf.

## Abbreviations

µL: Microliter
ANOVA: Analysis of variance
CASI-5: Child and Adolescent Symptom Inventory-edition 5
CD4: Cluster of differentiation 4
DNA: Deoxyribonucleic acid
DSM-5: Diagnostic and statistical manual of mental disorders-edition 5
GWAS: Genome-wide association
HIV/AIDS: Human immunodeficiency virus/Acquired immunodeficiency disease syndrome
HIV+: Human immunodeficiency virus-positive
HPA: Hypothalamic–pituitary–adrenal
HWE: Hardy–Weinberg equilibrium
IMD: Internalzing mental disorder
IMDs: Internalizing mental disorders
JCRC: Joint Clinical Research Council
MDD: Major depressive disorder
MRC/DfID: Medical Research Council/Department for International Development
ng: Nano gram
PTSD: Post-traumatic stress disorder
qPCR: Quantitative polymerase chain reaction
TL: Relative telomere length
RNA: Ribonucleic acid
s: Seconds
S: Single copy gene
SES: Socio-economic status
SNP: Single nucleotide polymorphism
SNPs: Single nucleotide polymorphisms
TASO: The AIDS Support Organization
TERC: Telomerase RNA component
TERT: Telomerase reverse transcriptase
TL: Telomere length
UVRI: Uganda Virus Research Institute
