# Supplementary material for: TERT rs2736100 and TERC rs16847897 genotypes moderate the association between internalizing mental disorders and accelerated telomere length attrition among HIV+ children and adolescents in Uganda
Source: BMC Med Genomics. 2021 Jan 6;14:15. doi: 10.1186/s12920-020-00857-z (PMC7789327; doi:10.1186/s12920-020-00857-z)
Supplement: Supplementary file 1 — Additional file 1. Figures S1 and S2 show the linkage disequilibrium map of TERT and TERC single nucleotide polymorphisms respectively. Table S1 shows the genotype and aplotype frequencies for TERT and TERC single nucleotide polymorphisms. [file 12920_2020_857_MOESM1_ESM.docx]

# Supplementary materials

**Figure S1**

LD plot showing linkage disequilibrium (LD) for *TERT* rs2736100, rs10069690, rs7726159 and rs2853669.


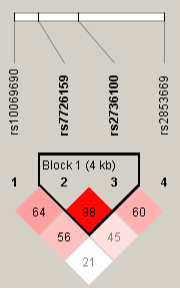


The linkage disequilibrium map of *TERT*. D’ values are depicted in the diamonds with darker colours depicting stronger LD. The LD map was created using the default Gabriel LD [67], implemented in Haploview, version 4.2 [68]. *TERT* rs7726159 and rs2736100 were in LD resulting in *CT*, *CG* and *AG*. Location = 5’ to 3’ on chromosome 5.

**Figure S2**


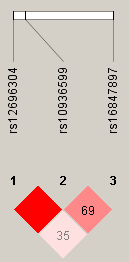


The linkage disequilibrium map of *TERC*. D’ values are depicted in the diamonds with darker colours depicting stronger LD. The LD map was created using the default Gabriel LD [67], implemented in Haploview, version 4.2 [68]. None of the SNPs were in LD. Location = 5’ to 3’ strand on chromosome 3.

**Table S1**: The genotype and haplotype frequencies for *TERT* and *TERC* single nucleotide polymorphisms

| Single nucleotide polymorphism/haplotype | Count (frequency) |
| --- | --- |
| *TERT* rs2736100 | |
| *GG* | 139 (0.21) |
| *TG* | 330 (0.49) |
| *TT* | 201 (0.30) |
| *TERT* rs7726159 | |
| *CC* | 417 (0.60) |
| *CA* | 245 (0.35) |
| *AA* | 31 (0.05) |
| *TERT* 10069690 | |
| *TT* | 286 (0.42) |
| *TC* | 317 (0.47) |
| *CC* | 73 (0.11) |
| *TERT* 2853669 | |
| *TT* | 522 (0.80) |
| *CT* | 125 (0.19) |
| *CC* | 8 (0.01) |
| *TERC* 12696304 | |
| *GG* | 237 (0.35) |
| *GC* | 352 (0.52) |
| *CC* | 87 (0.13) |
| *TERC* 16847897 | |
| *GG* | 373 (0.55) |
| *GC* | 265 (0.39) |
| *CC* | 44 (0.06) |
| *TERC* 10936599 | |
| *CC* | 650 (0.95) |
| *TC* | 32 (0.05) |
| *TT* | 0 (0) |
| *TERT* rs2736100-rs7726159 haplotype | |
| *CT* | 173 (0.77) |
| *CG* | 24 (0.11) |
| *AG* | 28 (0.12) |
